# Analysis on the green total factor productivity of pig breeding in China: Evidence from a meta-frontier approach

**DOI:** 10.1371/journal.pone.0270549

**Published:** 2022-06-24

**Authors:** Shen Zhong, Junwei Li, Xiangyu Guo

**Affiliations:** 1 School of Finance, Harbin University of Commerce, Heilongjiang, PR China; 2 School of Economics and Management, Northeast Agricultural University, Heilongjiang, PR China; Northeastern University (Shenyang China), CHINA

## Abstract

The pig industry occupies an extremely significant position in agriculture. The input cost, output income and the amount of pollution emitted by pig farming of different scales are unequal. It is of great practical importance to reduce pollutant emission by improving efficiency for the development of hog breeding industry in China. With the addition of undesirable output, this paper uses the Slack Based Measure- Metafrontier Malmquist Luenberger index model considering scale heterogeneity to explore the evolution characteristics of China’s green total factor productivity of pig breeding (GTPB) based on the data of China’s 17 major pig producing provinces from 2004 to 2018. The results indicate that: (1) From 2004 to 2018, China’s large-scale GTPB is the highest, the medium-sized is the second, and the small-scale is the lowest. (2) In terms of regional distribution, China’s GTPB in western region is the highest, in eastern region is the second, and in central region is the lowest. (3) China’s GTPB shows efficiency growth and technological decline from 2004 to 2018. The pig breeding industry is generally fragile, which is greatly affected by emergencies. (4)The TGR of large-scale pig breeding is closest to 1, followed by middle-scale, and finally small-scale. According to the above empirical results, this text puts forward some policy suggestions to improve GTPB and environmental protection recommendations of hog breeding.

## 1. Introduction

For a long time, the pig industry has been the most essential basic industry in China’s traditional agriculture. The stable development of the pig industry and the effective supply of pork are one of the people’s livelihood issues that the country is most concerned about [1, 2]. In 2020, the No.1 Document of the Central Committee of China pointed out that “Stabilizing the supply of live pigs is a major event in the current economic work. Comprehensive measures must be taken to ensure the stability of pork supply.” There are two ways to increase the supply of live pigs, one is to expand the scale of pig breeding [3], the other is to improve the production efficiency of pig breeding [4]. This paper aims to measure the total factor productivity of pig breeding, in order to obtain the way to improve the efficiency of pig production.

There are significant differences in the basic situation of pig breeding in different scales [5–7]. First of all, three scales exist difference in the number of pig farms, breeding costs, farming methods, yield and breeding profits [8, 9]. Secondly, infrastructure, rural human capital level, and environmental management level of breeding technology also produce diverse effects depending to the distinct scale of pig breeding [10–12]. Thirdly, the amount of manure and urine produced during pig breeding of unequal scales are different, thus the pollution emissions of chemical oxygen demand (COD), total nitrogen (TN) and total phosphorus (TP) converted from them are various [13–15]. It should be pointed out that while the pig industry has made considerable progress, it inevitably brings about environmental problems [16–18], The pollutants will cause a series of negative environmental impacts, such as air pollution, water pollution and soil pollution, and even endanger human and animal health [19–22]. Therefore, it is of great practical importance to reduce pollutant emission by improving efficiency for the development of hog breeding industry in China. This paper adds undesirable output and takes into account the scale heterogeneity, using Slack Based Measure- Metafrontier Malmquist Luenberger (SBM-MML) model to comprehensively evaluate the changes in green total factor productivity (GTFP) of China’s hog industry from 2004 to 2018. This is of great significance to the amelioration of China’s live pig supply and production efficiency, as well as the improvement of China’s environmental quality.

The second part of this paper introduces the research situation of relevant literature. The third part described the relevant theoretical basis and the source of indicators and data. The fourth part is empirical analysis. The conclusion and relevant policy recommendations are explained in the fifth part.

## 2. Literature review

Currently, there have some studies on the pig industry in other countries. Peters et al. (2005) studied the situation of finishing pigs in 13 provinces of Hanoi, Vietnam from 1997 to 2003 [23]. Hediger (2006) studied the pollution caused by pig breeding in Swiss farms from 2000 to 2010 [24]. It is found that the organic matter contained in animal feces was an important source of greenhouse gases. There are also some studies on China’s pig breeding industry, but the data span is short. Xu et al. (2015) studied China’s pig industry from 2004 to 2010 and found that labor output elasticity will show a downward trend over time. And the technical efficiency level in the eastern region is higher than that in the central region and the western region [25]. Subsequently, Du et al. (2017) studied the development of pigs in China from 2004 to 2014, and considered that the coastal and central regions are more suitable for developing small-scale and middle-scale breeding, the northeast region is more suitable for developing large-scale breeding, and the southwest region is more suitable for developing both small-scale and large-scale breeding [26]. The above scholars have made great contributions to the research of China’s pig breeding industry, but it can be seen that the previous study sample were not more than decade, and lacked research on large sample data of China’s pig breeding in recent years.

There are parameter method and nonparametric method to calculate the efficiency. Stochastic Frontier Analysis (SFA) is the main representative of parametric method, and Data Envelopment Analysis (DEA) is the main representative of nonparametric method. DEA can avoid the strong hypothesis bias of SFA about model form setting and random interference normal distribution, so it is more widely used. Brewer et al. (1998) used the traditional data envelopment analysis (DEA) model to calculate the fluctuation period of the year-end stock, pig output and pork production [27]. The principle of traditional DEA method is relatively simple, and it has special advantages for multi-input-output system. It does not need to preset the production function, so it is more applicable and convenient for the real economic system. However, when evaluating the efficiency of decision-making unit (DMU), this model does not take into account the impact of slack variables, which may lead to the deviation of efficiency measurement [28]. At the same time, the traditional DEA model is mainly based on the distance function that the expected output and the unexpected output expand in the same proportion, which means that the unexpected output cannot be reduced [29, 30]. The SBM model can make up for the shortcomings of traditional DEA, measure the invalid rate from the perspective of input and output, and adjust the input, expected output and unexpected output in different proportions [31, 32]. Its advantage is that it takes into account the “crowding” or “relaxation” of factors, and is a non-radial efficiency measurement method, which can better reflect the essence of environmental efficiency evaluation than other models [33]. The SBM model not only has stronger efficiency discrimination than traditional DEA model, but also can provide more redundant information related to input-output efficiency [34]. Referring to the existing literature and considering these advantages, this paper uses SBM model to measure the GTPB. However, it’s a long-term continuous process. The SBM model can only measure the static efficiency of a certain time section, and can be used to horizontally compare the GTFP of 17 major pig producing provinces in China. In the past, malmquist total factor productivity (TFP) index method was used to analyze the productivity changes of production DMUs from multiple time points. Fan and Zhang (2002) adopted the dynamic nonparametric DEA-Malmquist method, based on the distance function, through the linear programming method to construct the multi-dimensional output [35]. Constructing multiple frontiers cannot strip the influence of stochastic effects on output. Goodland and Anhang (2009) used Malmquist- Luenberger method to point out the Agriculture Committee mistakenly attributed the emission sources belonging to the animal husbandry sector to other sectors. That is to say, the world’s annual carbon dioxide emissions from animal husbandry and animal husbandry by-products production are huge, accounting for more than half of the world’s total carbon dioxide emissions. The study points out the seriousness of the current situation of greenhouse in animal husbandry, calls on all countries to strengthen the low-carbon development of animal husbandry and promotes energy conservation and emission reduction [36]. Likewise, Chamniansawat and Chongthammakun (2012) used Malmquist-Luenberger method to study the treatment methods of three main livestock breeding wastes. The results showed that the conversion of waste into biogas could improve the overall economic benefits. But if we wanted to obtain greater economic benefits, the generated biogas could be sold [37]. Hayami (1969) and Hayami and Ruttan (1971) first proposed the concept of common frontier, which is more suitable for examining the input-output relationship among different categories at the same time [38, 39]. Based on the number of sample units and data characteristics, combined with the advantages of SBM model, this text uses SBM-MML model to vertically compare the changes in GTFP from the time dimension. The previous efficiency evaluation models usually put all DMUs together to construct the frontier, and analyze the efficiency by measuring the distance between each individual and the frontier. Thus, it is generally considered to be without distinguishing the heterogeneity of DMUs [40]. In fact, there is heterogeneity in GTPB under different farming scales. If all DMUs are still assumed to face the same or similar technical boundaries to evaluate the GTFP of the overall sample, the results will be biased. The common frontier theory breaks the traditional homogeneity assumption and focuses on exploring the heterogeneity characteristics of DMUs [41–43]. The current research on pig breeding industry does not consider the importance of scale heterogeneity for the study of GTPB under the meta-frontier.

Under the background of the increasingly perfect relevant environmental protection policies and gradually strengthened environmental regulations in China, how to improve the efficiency of pig breeding under environmental high pressure policy has become an important issue at present. Yang et al. (2003) and Zhou et al. (2007) were only limited to the statistical description and calculation of pig pollutant emissions, which to some extent separated the close relationship between pig breeding efficiency and environment [44, 45]. However, with the gradual tightening of environmental constraints and the acceleration of the sustainable development process in pig breeding industry, the combination of environmental problems and TFP of pig breeding is undoubtedly an effective way to more truly reflect the actual production. Improving the GTFP is the key to achieve a win-win development of pollution control and pig production. Therefore, to solve the fundamental problem of environment and pig breeding is to comprehensively improve the GTFP. In recent years, some scholars have tried to consider the resource and environmental constraints to study China’s pig industry. Sharma et al. (1997) only considered labor, capital and other traditional factors to calculate the TFP of pig breeding [46]. Reinhard et al. (2000) took environmental harmful substances as output variables, namely excess nitrogen, excess phosphorus and direct and indirect energy consumption, and calculated the scores of GTFP in Dutch live pig [47]. By comparing the scores of average efficiency and comprehensive efficiency, the advantages and disadvantages of various methods were evaluated effectively, and effective measures were put forward to improve the comprehensive environmental efficiency. From June 1998 to February 2001, Kliebenstein et al. (2003) conducted a comparative study on pig growth completion facilities at Ross Research and Demonstration Farm of Iowa State University in the United States [48]. Selecting piglet weight, feed price and market pork price as variables, it is considered that the variable cost of large-scale pig farming is smaller than that of small-scale pig breeding. The utilization rate of feed and human cost, management skills and breeding technology of large-scale pig farming are higher, so the benefit of large-scale breeding is much better than that of small-scale pig farming. Ball et al. (2004) used the environmental index of carbon emission to measure the environmental TFP of pigs. The results showed that the environment had a significant negative inhibitory effect on the TFP of live pig, and the environmental TFP had obvious differences between scales and regions [49]. Kumar (2006) believed that with the pollution problem gradually aroused people’s attention, it is a more comprehensive analysis frame to incorporate environmental factors into the production efficiency analysis framework by using different models [50]. Liu et al (2011) and Alexiadis (2012) measured carbon emissions, total pollutants, chemical oxygen demand, total nitrogen and total phosphorus as environmental indicators, and concluded that China’s pig breeding industry has not yet reached the stage of sustainable development of ecological environment, ignoring environmental pollution factors will overestimate GTPB [51, 52]. Besides, the stable growth of GTPB is supported by technological progress, and the technical efficiency has been running at a low level. Bai et al (2014) established a multi factor model to calculate nitrogen loss in pig breeding [53]. From the study of the above scholars can be seen, the existing research for pig breeding input and output index selection is relatively single, and it does not take into account the different weights given to pollutants for conversion.

To sum up, there are many deficiencies in the existing research on pig efficiency, and the calculation of TFP considering environmental factors is still relatively small, Most studies only simply sum up the emissions of COD, TN, TP and other pollutants, without considering the weighting problem, so the validity of the calculation results has a large error. The research focusing on the perspective of scale grouping is rarer, and most of them only stay at the level of efficiency measurement and decomposition. Therefore, the objective of this paper is mainly reflected in the following aspects: (1) In terms of sample and data selection, this paper selects the data of 17 major pig producing areas in China from 2004 to 2018, and the samples are more representative and the data are more contemporary. (2) In the model construction, the SBM-MML model is used to comprehensively analyze the development of GTPB under different scales through the comparison of common frontier and scale frontier. (3) In the variable selection and index calculation, the negative output is added, and the COD, TN and TP are given different weights to calculate the total pollution emissions. In order to provide more reliable theoretical basis and environmental policy recommendations for the government’s decision-making on the development of pig breeding industry.

## 3. Methodology

### 3.1 SBM model based on common frontier

Tone (2001) proposed Slack Based Measure (SBM) model [54], its calculation principle is as Eq (1).


$$\begin{array}{l} \min\rho =\frac{1-\frac{1}{M}\sum_{m=1}^{M}\frac{s_{m}^{x}}{x_{m0}}}{1+\frac{1}{N}\sum_{n=1}^{N}\frac{s_{n}^{y}}{y_{n0}}}\\ s.t.\left\{ \begin{array}{l} \sum_{k=1}^{K}\theta x_{km}+s_{m}^{x}=x_{m0},m=1,\cdots ,M;\\ \sum_{k=1}^{K}\theta y_{kn}-s_{n}^{y}=y_{n0},n=1,\cdots ,N;\\ \theta \geq 0;s_{m}^{x}\geq 0;s_{n}^{y}\geq 0 \end{array}\right. \end{array}$$ (1)

Where *ρ* represents the efficiency value of the evaluated DMU, which analyzes the invalid rate from two aspects of input and output, so it is called non-angle model. If *ρ* is equal to 1, the DMU is strong efficient and there is no weak efficiency problem in the radial model. *M* and *N* are the number of input variables and expected output variables, respectively. The subscript 0 represents a DMU to be solved, and *θ* is the respective weight of DMU *k* in the construction of environmental technology structure. $\left(S_{m}^{x},S_{n}^{y}\right)$ is the slack of input redundancy and insufficient expected output. *x* and *y* are the input and expected output vectors, respectively. Cooper et al. (2007) combined SBM model with environmental technology to establish SBM model considering environmental factors [55], it can be written as Eq (2).


$$\begin{array}{l} \min\rho =\frac{1-\frac{1}{M}\sum_{m=1}^{M}\frac{s_{m}^{x}}{x_{m0}}}{1+\frac{1}{N+I}(\sum_{n=1}^{N}\frac{s_{n}^{y}}{y_{n0}}+\sum_{i=1}^{I}\frac{s_{i}^{b}}{b_{i0}})}\\ s.t.\left\{ \begin{array}{l} \sum_{k=1}^{K}\theta x_{km}+s_{m}^{x}=x_{m0},m=1,\cdots ,M;\\ \sum_{k=1}^{K}\theta y_{kn}-s_{n}^{y}=y_{n0},n=1,\cdots ,N;\\ \sum_{k=1}^{K}\theta b_{ki}+s_{i}^{b}=b_{i0},i=1,\cdots ,I;\\ \theta \geq 0;s_{m}^{x}\geq 0;s_{n}^{y}\geq 0;s_{i}^{b}\geq 0 \end{array}\right. \end{array}$$ (2)

Where *M*, *N* and *I* are the number of input variables, expected output variables and unexpected output variables, respectively. The subscript 0 is a DMU to be solved, and the *θ* is the weight of DMU *k* in the construction of environmental technology structure. $\left(S_{m}^{x},S_{n}^{y},S_{i}^{b}\right)$ represents the slack of input redundancy, expected output insufficiency and unexpected output redundancy. *x*, *y* and *b* are input vector, expected output vector and unexpected output vector, respectively. The higher the efficiency value *β* of the objective function is, the higher the efficiency value of evaluating DMU is. The efficiency value of 1 indicates that the evaluation of DMU is strongly effective.

**Fig 1:**
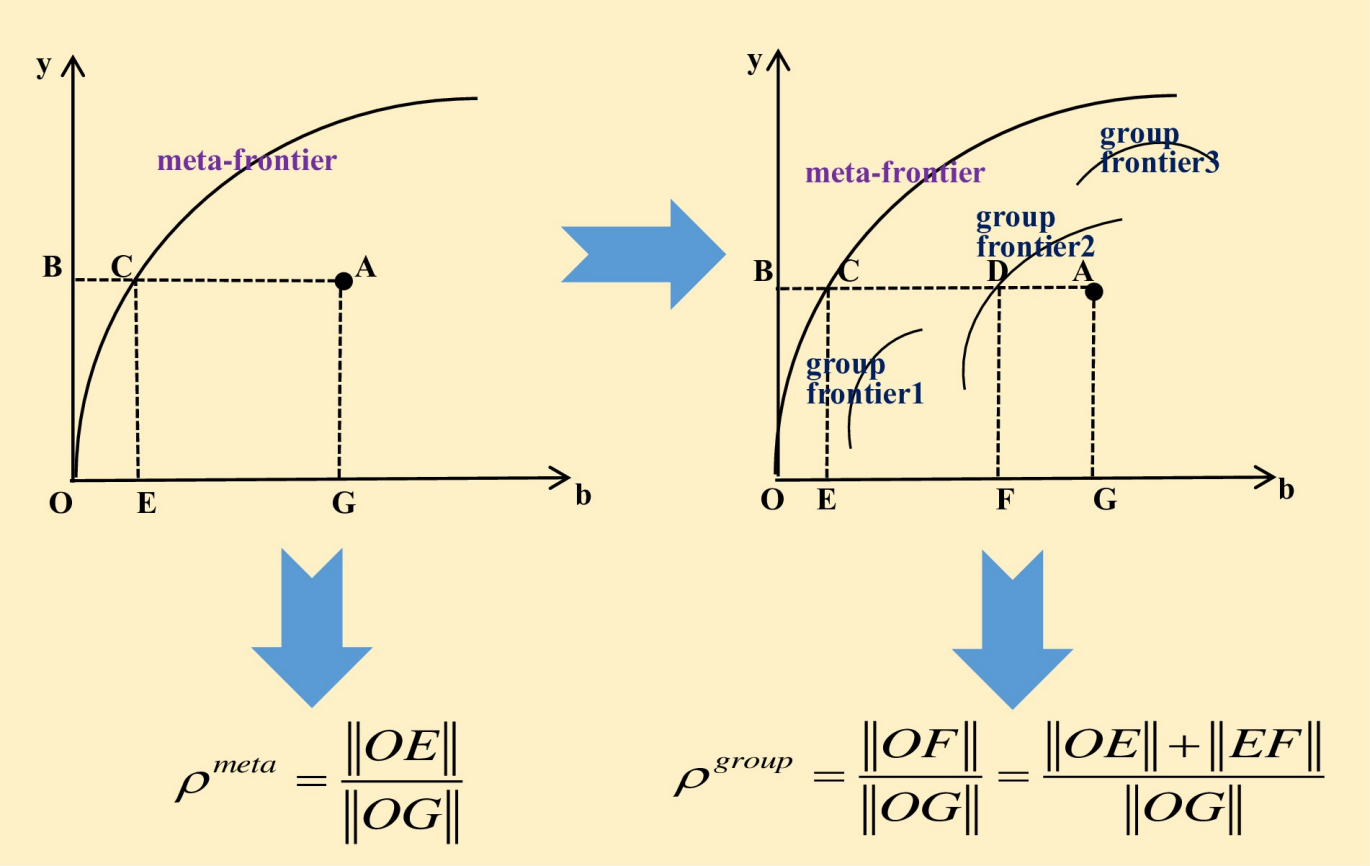
Structure chart of common frontier and scale frontier.

DEA is a non-parametric method, which can’t calculate the TFP of two periods, but its TFP index can be calculated, that is $MI_{t-1}^{t}$. Taking all input-output data of the sample period as the reference set of the current period, the global DEA method is used to construct the production frontier, and calculate China’s GTPB and its decomposition index under the conditions of common frontier and group frontier, respectively. According to Guo et al. (2017) [56], as shown in Fig 1, the analysis framework of common boundary and group boundary (scale boundary) is constructed to investigate GTPB under different scale boundary. The GTPB of DMU A under the meta-frontier and the intergroup frontier can be defined as Eqs (3)–(4):

$$\rho^{meta}=\frac{\left\|OE\right\|}{\left\|OG\right\|}$$ (3)

$$\rho^{group}=\frac{\left\|OF\right\|}{\left\|OG\right\|}=\frac{\left\|OE\|+\|EF\right\|}{\left\|OG\right\|}$$ (4)

And the value of *ρ*^*meta*^ and *ρ*^*group*^ can be gained by solving following DEA model (Eq (5) and Eq (6)):

$$\begin{array}{l} \min\rho^{meta}=\frac{1-\frac{1}{M}\sum_{t=1}^{T}\sum_{m=1}^{M}\frac{s_{m}^{x}}{x_{m0}^{t}}}{1+\frac{1}{N+I}(\sum_{t=1}^{T}\sum_{n=1}^{N}\frac{s_{n}^{y}}{y_{n0}^{t}}+\sum_{t=1}^{T}\sum_{i=1}^{I}\frac{s_{i}^{b}}{b_{{}_{i0}}^{t}})}\\ s.t.\left\{ \begin{array}{l} \sum_{t=1}^{T}\sum_{k=1}^{K_{M}}\mu^{t}x_{{}_{km}}^{t}+s_{m}^{x}=x_{{}_{m0}}^{t},m=1,\cdots ,M;\\ \sum_{t=1}^{T}\sum_{k=1}^{K_{M}}\mu^{t}y_{{}_{kn}}^{t}-s_{n}^{y}=y_{{}_{n0}}^{t},n=1,\cdots ,N;\\ \sum_{t=1}^{T}\sum_{k=1}^{K_{M}}\mu^{t}b_{{}_{ki}}^{t}+s_{i}^{b}=b_{{}_{i0}}^{t},i=1,\cdots ,I;\\ \mu^{t}\geq 0;s_{m}^{x}\geq 0;s_{n}^{y}\geq 0;s_{i}^{b}\geq 0;t=1,\cdots ,T;k=1,\cdots ,K_{M} \end{array}\right. \end{array}$$ (5)

$$\begin{array}{l} \min\rho^{group}=\frac{1-\frac{1}{M}\sum_{t=1}^{T}\sum_{m=1}^{M}\frac{s_{m}^{x}}{x_{m0}^{t}}}{1+\frac{1}{N+I}(\sum_{t=1}^{T}\sum_{n=1}^{N}\frac{s_{n}^{y}}{y_{n0}^{t}}+\sum_{t=1}^{T}\sum_{i=1}^{I}\frac{s_{i}^{b}}{b_{{}_{i0}}^{t}})}\\ s.t.\left\{ \begin{array}{l} \sum_{t=1}^{T}\sum_{k=1}^{K_{G}}\lambda^{t}x_{{}_{km}}^{t}+s_{m}^{x}=x_{{}_{m0}}^{t},m=1,\cdots ,M;\\ \sum_{t=1}^{T}\sum_{k=1}^{K_{G}}\lambda^{t}y_{{}_{kn}}^{t}-s_{n}^{y}=y_{{}_{n0}}^{t},n=1,\cdots ,N;\\ \sum_{t=1}^{T}\sum_{k=1}^{K_{G}}\lambda^{t}b_{{}_{ki}}^{t}+s_{i}^{b}=b_{{}_{i0}}^{t},i=1,\cdots ,I;\\ \lambda^{t}\geq 0;s_{m}^{x}\geq 0;s_{n}^{y}\geq 0;s_{i}^{b}\geq 0;t=1,\cdots ,T;k=1,\cdots ,K_{G} \end{array}\right. \end{array}$$ (6)

Where T is the number of years; K_M_ and K_G_ represent the number of DMUs under the common frontier and group frontier, respectively; μ and λ are the intensity variables under the common frontier and group frontier, respectively; and production possibility is under assumption of the constant returns to scale (CRS).

### 3.2 Metafrontier-Malmquist-Luenberger index and its decomposition

The Malmquist index was first proposed by Swedish scholar Malmquist in 1952 to analyze consumption changes in different periods. Chung et al. (1997) applied the directional distance function containing undesirable outputs to the Malmquist model, and made the obtained Malmquist index as Malmquist-Luenberger (ML) index [57]. At this point, any Malmquist index with unexpected output can be called Malmquist-Luenberger index. ML index belongs to Malmquist index. On this basis, Oh (2010) constructed the Metafrontier-Malmquist-Luenberger (MML) index [58]. All the evaluated DMUs are included in the global reference set, which is expressed as Eqs (7)–(8):

$$P^{G}(x)=P^{1}(x^{1})\cup P^{2}(x^{2})\cup \ldots \cup P^{T}(x^{T})$$ (7)

$$P^{t}(x^{t})=\{\left(y^{t},b^{t})|x^{t}\;can\;produce\;(y^{t},b^{t})\right\}$$ (8)

The change of GTPB is analyzed from a global perspective. This paper selects MML index as GTPB. At the same time, in order to further explore the sources of GTPB changes, this text further decomposes MML index into efficiency change (EC) index and technology change (TC) index. EC mainly refers to the improvement of resource allocation efficiency and management system, and TC refers to the amelioration of production technology. MML takes the sum of all periods as a reference, and each period refers to the same frontier. According to Pastor and Lovell (2005) [59], the MML index formula is as Eq (9):

$$\begin{array}{l} MML_{t-1}^{t}=\frac{\rho^{G}(x^{t},y^{t},b^{t};y^{t},-b^{t})}{\rho^{G}(x^{t-1},y^{t-1},b^{t-1};y^{t-1},-b^{t-1})}\\ =\left[\frac{\rho^{G}(x^{t},y^{t},b^{t};y^{t},-b^{t})}{\rho^{t}(x^{t},y^{t},b^{t};y^{t},-b^{t})}/\frac{\rho^{G}(x^{t-1},y^{t-1},b^{t-1};y^{t-1},-b^{t-1})}{\rho^{t-1}(x^{t-1},y^{t-1},b^{t-1};y^{t-1},-b^{t-1})}\right]\\ \times \frac{\rho^{t}(x^{t},y^{t},b^{t};y^{t},-b^{t})}{\rho^{t-1}(x^{t-1},y^{t-1},b^{t-1};y^{t-1},-b^{t-1})}=TC_{t-1}^{t}\times EC_{t-1}^{t} \end{array}$$ (9)

Where the input, expected output and unexpected output of t-1 and t are expressed as $\left(x^{t-1},y^{t-1},b^{t-1}\right)$ and $\left(x^{t},y^{t},b^{t}\right)$, respectively; $\rho^{G}(x^{t},y^{t},b^{t};y^{t},-b^{t})$ and $\rho^{G}(x^{t-1},y^{t-1},b^{t-1};y^{t-1},-b^{t-1})$ denote the GTPB of DMUs from period t-1 to period t defined on *P*^*G*^; $TC_{t-1}^{t}$ and $EC_{t-1}^{t}$ represent the contribution to GTPB promotion of DMU’s technological progress and efficiency improvement, respectively, from t-1 to t. The larger the value is, the greater the contribution is. The MML index is denoted as MI.

For the DMUs with heterogeneous scale, we can calculate the scale gap between the group frontier and the common frontier, which is caused by the specific group institutional structure. The basic idea of this method is to construct the common boundary and group boundary under the same input factors, and the environmental efficiency values under the common frontier and the group frontier are calculated respectively. Then the technology gap ratio (TGR) of DMU under the meta-frontier and group frontier is obtained. The calculation formula is as Eq (10):

$$TGR=\frac{\rho^{meta}}{\rho^{group}}=\frac{\left\|OE\right\|}{\left\|OF\right\|}$$ (10)

Where *ρ*^*meta*^ and *ρ*^*group*^ are the environmental efficiency value under the meta-frontier and scale frontier, respectively. Obviously, 0≤*TGR*≤1. TGR is used to measure the distance between the optimal production scale and the potential optimal scale of the group. The closer the TGR is to 1, the closer the scale level is to the optimal potential scale level. On the contrary, that is the greater the gap between the scale level and the potential optimal scale level.

### 3.3 Description and sources of data

Based on the existing literature, this paper selects five indicators to construct the input-output index system. The details are as follows:

1. Piglet input: Piglet weight. It refers to the average weight of each piglet purchased from outside or bred by their own pigs.
2. Labor input: The number of labors. It is the sum number of direct labor days of agricultural employees and family employment.
3. Cost input: That is, cost input, containing water and fuel power cost, concentrate feed cost, medical and epidemic prevention cost. The cost of water and fuel power contains the cost of water, coal, electricity and other fuel power; the cost of concentrate feed refers to the input cost of various kinds of concentrate feed actually consumed by each pig from purchase to fattening and marketing, containing the total cost of mixed feed, grain, plant powder, bean cake, etc.; the cost of medical and epidemic prevention contains the cost of disease prevention and control.
4. Positive output: The output of main products, which refers to the live weight of each finishing pig at the time of marketing.
5. Negative output: Total pollutant discharge.

According to the calculation formula of Du et al. (2017) [26], the concrete calculation procedures are as Eqs (11)–(13):

$$CDA=AFD\times CDC\times \frac{AW}{RW}$$ (11)

$$NDA=AFD\times NDC\times \frac{AW}{RW}$$ (12)

$$PDA=AFD\times PDC\times \frac{AW}{RW}$$ (13)

Where CDA, NDA and PDA are the COD discharge amount, the TN discharge amount, and the TP discharge amount, respectively. CDC, NDC and PDC represent the COD discharge coefficient, the TN discharge coefficient, and the TP discharge coefficient. AFD is the average feeding days. AW and RW represent the actual weight and reference weight. According to the calculation formula of Zuo et al. (2016) [60], the calculation formula of total pollutant discharge is as Eq (14):

$$TPD=\frac{CDA}{20}+\frac{NDA}{1}+\frac{PDA}{0.2}$$ (14)

Where *TPD* is the total pollutant discharge.

Piglet weight, number of labors, cost of water and fuel, cost of concentrate feed, medical expenses, average feeding days, actual weight and main product yield are all from 2004–2018 *“National Compendium of Agricultural Product Expenses-Benefit Data”*. The pollution discharge coefficient and reference weight are from the manual of pollution discharge coefficient issued by the leading group office of the first national pollution source survey. The proportion of dry and clean feces and water washed feces in each province is referred to Du et al. (2017) [26]. At the same time, according to the definition of scale in the above two data, this paper divides pig breeding scale into three types: small scale, medium scale and large scale. Large scale refers to the breeding area with annual output of more than 1000 pigs; medium scale refers to scale farms with annual output of 100–1000 pigs; and small-scale refers to breeding specialized households with annual output of 30–100 heads.

In terms of sample selection, this paper selects 17 major pig producing provinces in the “National Pig Production Development Plan (2016–2020)” as research samples. It is divided into three regions: Eastern Region (Liaoning, Hebei, Jiangsu, Zhejiang, Shandong, Guangdong), central region (Jilin, Henan, Anhui, Heilongjiang, Hubei, Hunan), and western region (Sichuan, Guizhou, Guangxi, Chongqing, Yunnan).

## 4. Results and discussions

### 4.1 China’s temporal dynamic change of GTPB

**Fig 2:**
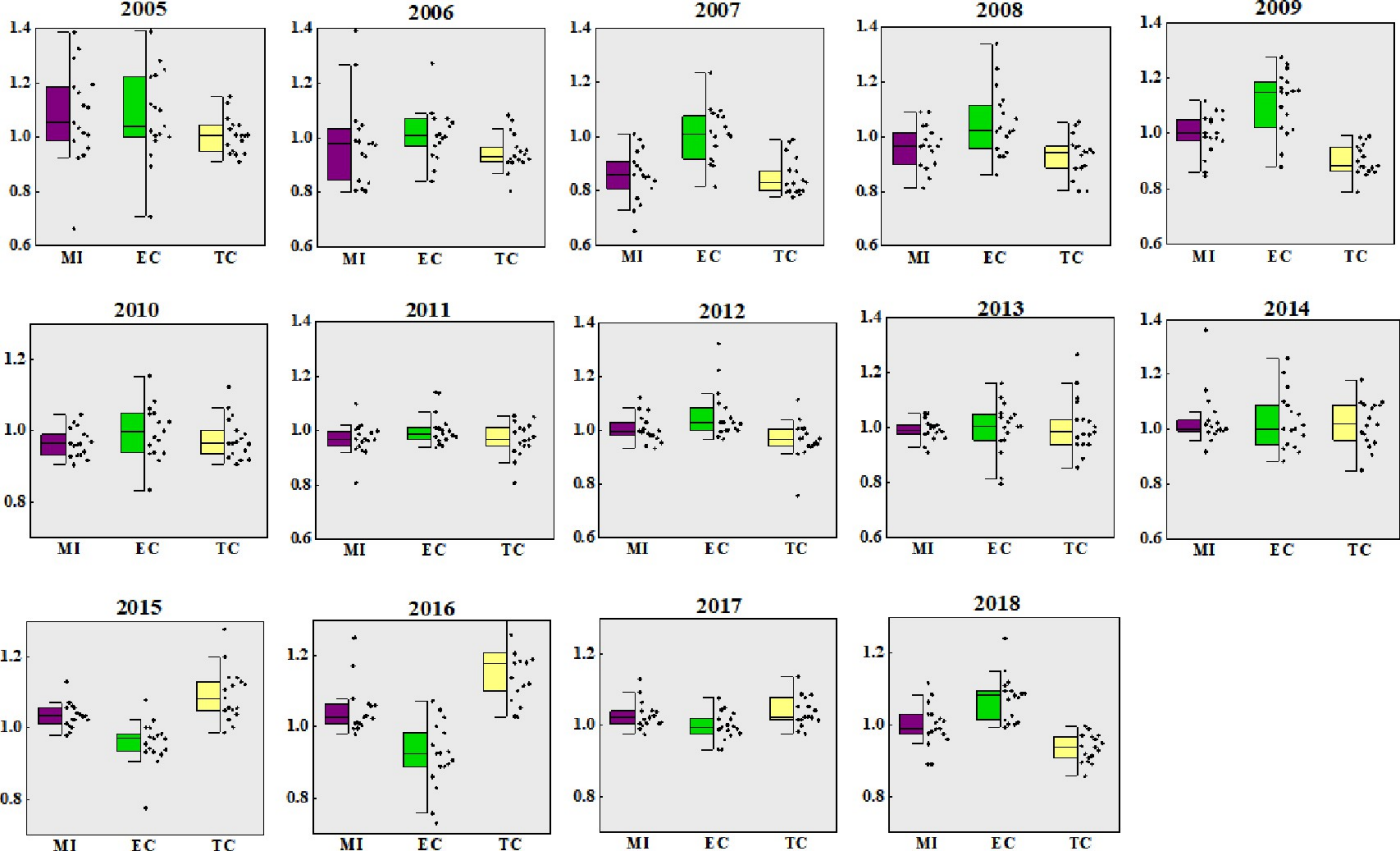
GTPB and its decomposition index under common frontier from 2004–2018.

Under the common frontier, the three-scale synthesis GTPB and their decomposition index (EC and TC) from 2004 to 2018 are shown in Fig 2.

It can be seen from Fig 2 that under the common frontier, the overall gap of China’s GTPB narrowed from 2005 to 2018. The distribution of values among each province becomes more and more intensive. The whole development situation was not very well from 2006 to 2010, and GTPB was lower than 1. This was mainly caused by the outbreak of blue ear disease in pigs in 2006, the global financial crisis in 2008, and the outbreak of influenza A (H1N1) virus in 2009. Moreover, in most years, the value of EC is higher than the TC, but in 2015–2017, the value of TC is higher than EC, for three consecutive years of technological progress. It indicates that with the development of economy, people’s living standards and the demand for pork continue to improve, and the pollution is also increasing. The government began to pay more attention to the emission of pollutants in the process of pig breeding. From 2015, In addition to carrying out related aspects of technological innovation and adopting advanced fecal treatment technology to reduce part of the environmental loss, China began to increase investment in low-carbon breeding technology and personnel training. However, the EC in 2018 is higher than TC, which may be because the previous three years have been sufficient investment in technology. The pig low-carbon breeding technology has gradually matured, and there is no longer too much for excessive investment of capital and talents. The top priority is to give full play to the utilization level of existing technologies, so that advanced technologies to play a greater role. Therefore, the EC is higher than the TC, and greater than 1. The low overall technical level and the large gap among provinces are the leading causes for the low GTPB in China. Improving the technical level and narrowing the technical gap between regions are important ways to effectively improve GTPB.

**Fig 3:**
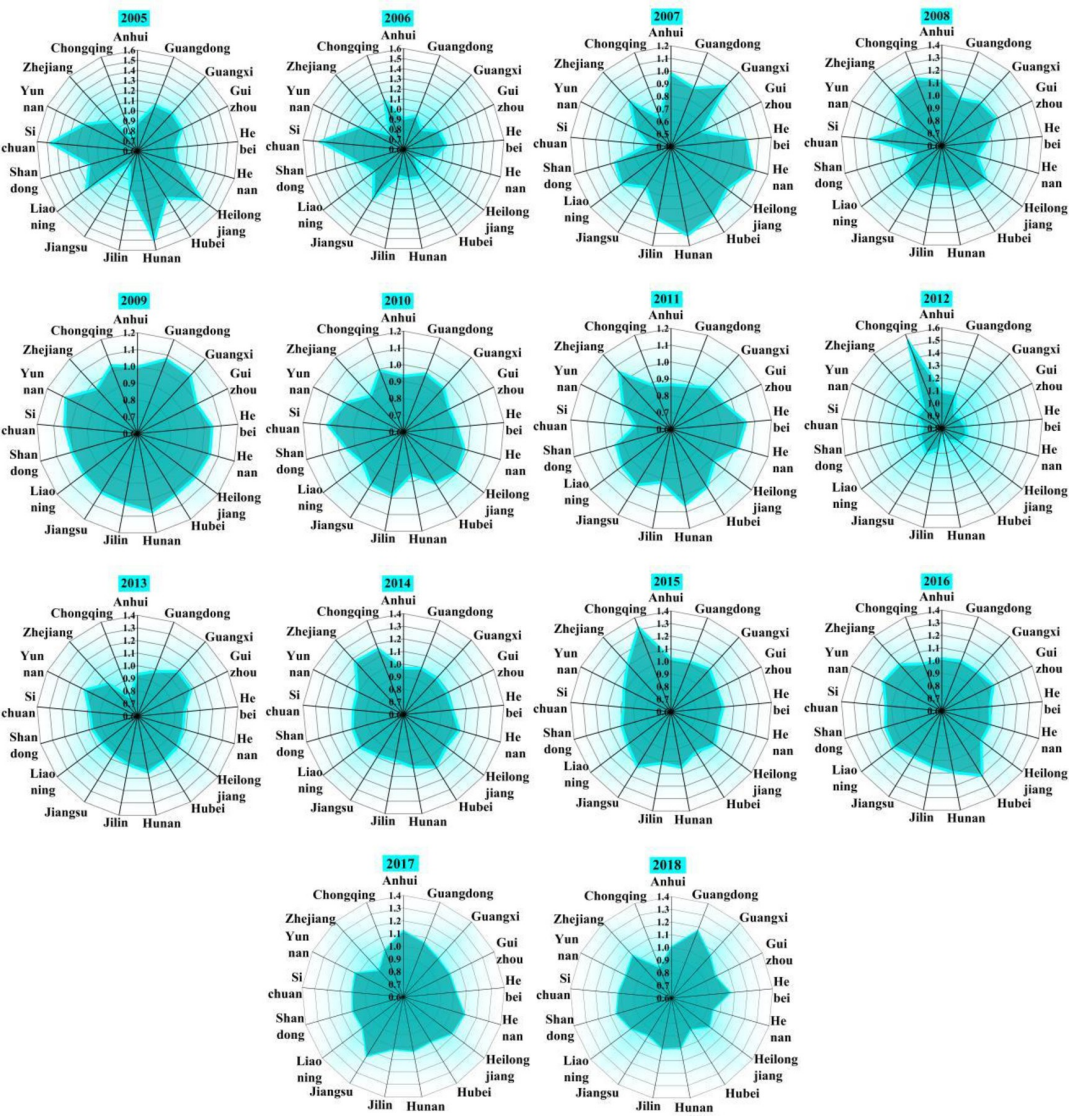
GTPB and its decomposition index under scale-frontier from 2004–2018.

Fig 3 displays three-sized integrated GTPB from 2005 to 2018 under the group frontier (scale-frontier). It can be seen from Fig 3 and Table 1 that the overall GTPB level in 2005 and 2016 under the group frontier is higher, and it has a trend of decline in recent years. Since 2012, the level of technology began to rise, mainly because China entered a period of intensive environmental protection policy (The “Twelfth Five Year Plan” period). In 2012, the “12th Five Year Plan of national livestock and poultry breeding pollution prevention and control” systematically summarized and analyzed the current situation, problems and facing forms of pollution prevention and control about livestock and poultry breeding in China. It provides scientific guidance for the various regions, and the prevention and control of environmental pollution of China’s animal husbandry has entered a new stage of development. In 2013, the State Council issued the first regulatory document "Regulations on pollution control of livestock and poultry scale breeding", specifically aimed at the prevention and control of livestock and poultry pollution. It was clear that the development planning of animal husbandry should take into account the environmental carrying capacity and the requirements for pollution prevention and control of livestock and poultry pollution, make a reasonable layout, scientifically determine the varieties, scale and total amount of livestock and poultry breeding, and at the same time clarify the criteria for the division of prohibited breeding areas. In 2015, the Ministry of Agriculture of China issued the “Guiding opinions on promoting the adjustment and optimization of pig breeding layout in the southern water network area” to guide the transfer of pig breeding to non-overloading areas and transfer production capacity from south to north, for the purpose of protecting the southern water network. In 2018, the Ministry of agriculture and Rural Affairs issued the policy of banning and limiting breeding, which forced the elimination of inferior farmers and farms with "low efficiency and high pollution", and optimized the pig breeding structure to a certain extent, which made the improvement of GTPB have certain developing space.

**Table 1:** EC and TC value under scale-frontier from 2004–2018.

	**2005**	**2006**	**2007**	**2008**	**2009**	**2010**	**2011**
GEC	1.0316	1.0370	0.9976	1.0422	1.0914	1.0092	1.0188
GTC	1.0767	0.9547	0.8987	0.9567	0.9280	0.9746	0.9615
	**2012**	**2013**	**2014**	**2015**	**2016**	**2017**	**2018**
GEC	1.0187	1.0160	1.0072	1.0014	0.9393	1.0081	1.0518
GTC	0.9995	0.9826	1.0510	1.0479	1.1635	1.0286	0.9576

In addition, since 2013, with the continuous decline in pork prices, the EC has continued to decline, falling below 1 in 2016. Based on the average data over the past 15 years, MI, EC and TC under the common frontier are 0.9982, 1.0223 and 0.9880 respectively, and 1.0052, 1.0193 and 0.9987 respectively, under the scale frontier. The results under the common frontier and the group frontier are different, which is mainly because the group frontier is based on different sizes of pig breeding structure frontier, while the common frontier is based on all scales of pig breeding structural frontier. The GTPB under the group frontier is obviously overestimated.

**Fig 4:**
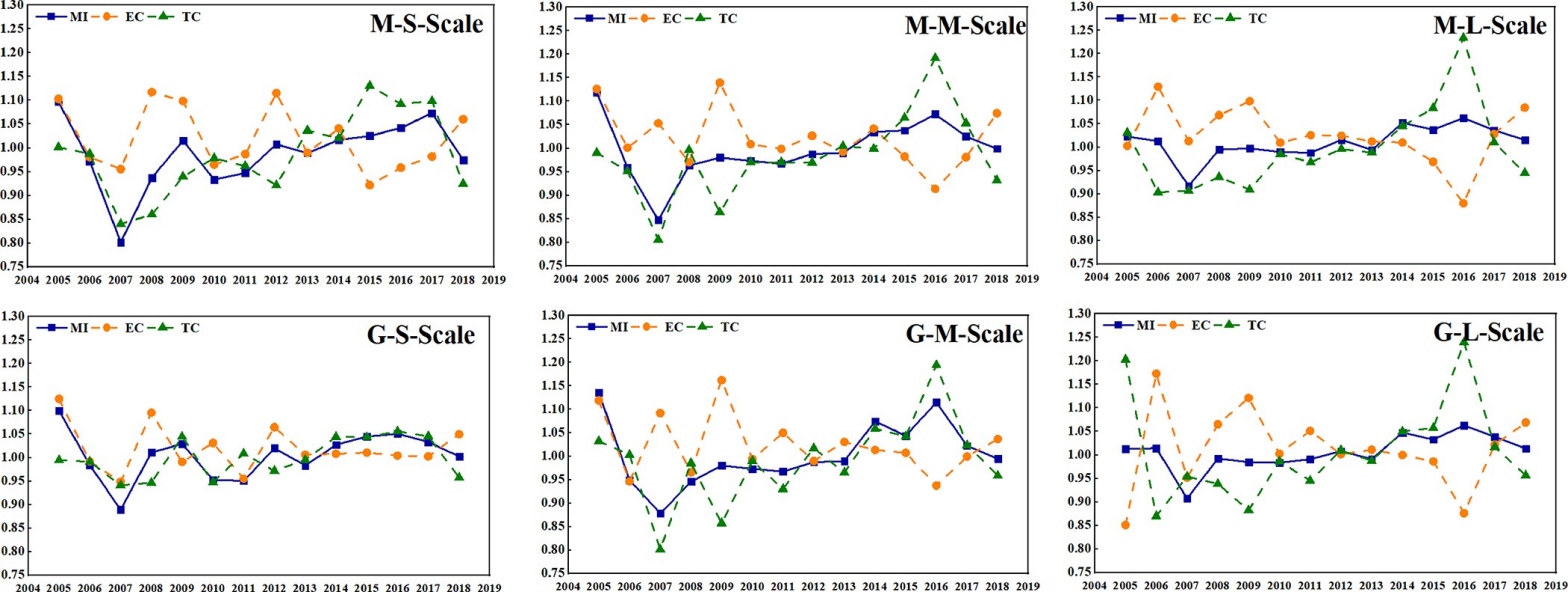
Three-sized GTPB and its decomposition index from 2004–2018.

It can be seen from Fig 4 that the change trends of GTPB under the common frontier and the group frontier are basically the same. The fluctuation range of small-scale GTPB is the largest, the second is medium-sized GTPB, and the smallest is large-scale GTPB, indicating that the development of large-scale pig breeding is the most stable.

Small-scale GTPB represented synchronous characteristics with the “pig cycle”. It was greatly affected by emergencies from 2004 to 2010. From 2010 to 2016, the development and popularization of technology level, the stricter policies related to environmental protection, and consumers’ pursuit of environmental quality all had a great impact on China’s pig breeding industry. In 2017, in the face of strict environmental supervision at the national level, the operation of environmental protection and pollution control facilities in various breeding sites increased the cost investment, reduced the investment in science and technology, and ignored the technological innovation, resulting in varying degrees of decline in TC and MI. Although the small-scale aquaculture has less pollution emissions, it has relatively weak pollutant treatment capacity and does not have a flexible manure return capacity, therefore small-scale farming is at a relative disadvantage position.

The level of medium-scale efficiency reached the maximum in 2009, then gradually decreased, and reached the minimum in 2016. On the contrary, the technical level was the minimum in 2009, and then increased continuously and reached the maximum value in 2016. It indicates that with the development and progress of society, the government and breeding personnel begin to pay attention to the investment and application of clean technology in pig breeding, and coordinate the relationship with nature while developing agriculture.

The large-scale fluctuation is relatively stable, because large-scale pig farming is greatly affected by policies and technologies, so it is not synchronized with the fluctuation of “pig cycle” as on a small-scale. Large-scale pig breeding has a strong ability to deal with pollutants. However, due to its large total emission, it also has strict requirements on equipment, labor force and professional technical level. Therefore, large-scale pig farming is characterized by the coexistence of high-tech efficiency and low resource utilization rate. The larger the scale is, the more advantages the grow of GTPB will be. In terms of the growth trend, the impact of environmental constraints on China’s pig breeding is greater, and the growth rate of GTPB is slowing down.

The GTPB of the three scales was the highest in2004, and the GTPB of the rest period was less than the initial period, which was mainly because the efficiency level of pig breeding had a downward trend under the background of the rising cost of pig breeding and the fluctuation of pork price. From 2005 to 2006, GTPB decreased by the largest margin in nearly 15 years. It can be seen that the blue ear disease epidemic in 2006 had a huge impact on China’s pig breeding industry, leading to at least five years of China’s pig breeding industry gradually returned to normal. Based on the above analysis, there are great differences in GTPB between different scale pig farming under the common frontier and the group frontier, which fully demonstrates the scientific and necessary to consider the scale heterogeneity in the analysis of GTPB. The reason for this phenomenon may be that distinct types of cities have different development paths such as industrial structure and resource allocation due to their various urban functions positioning and economic development.

**Fig 5:**
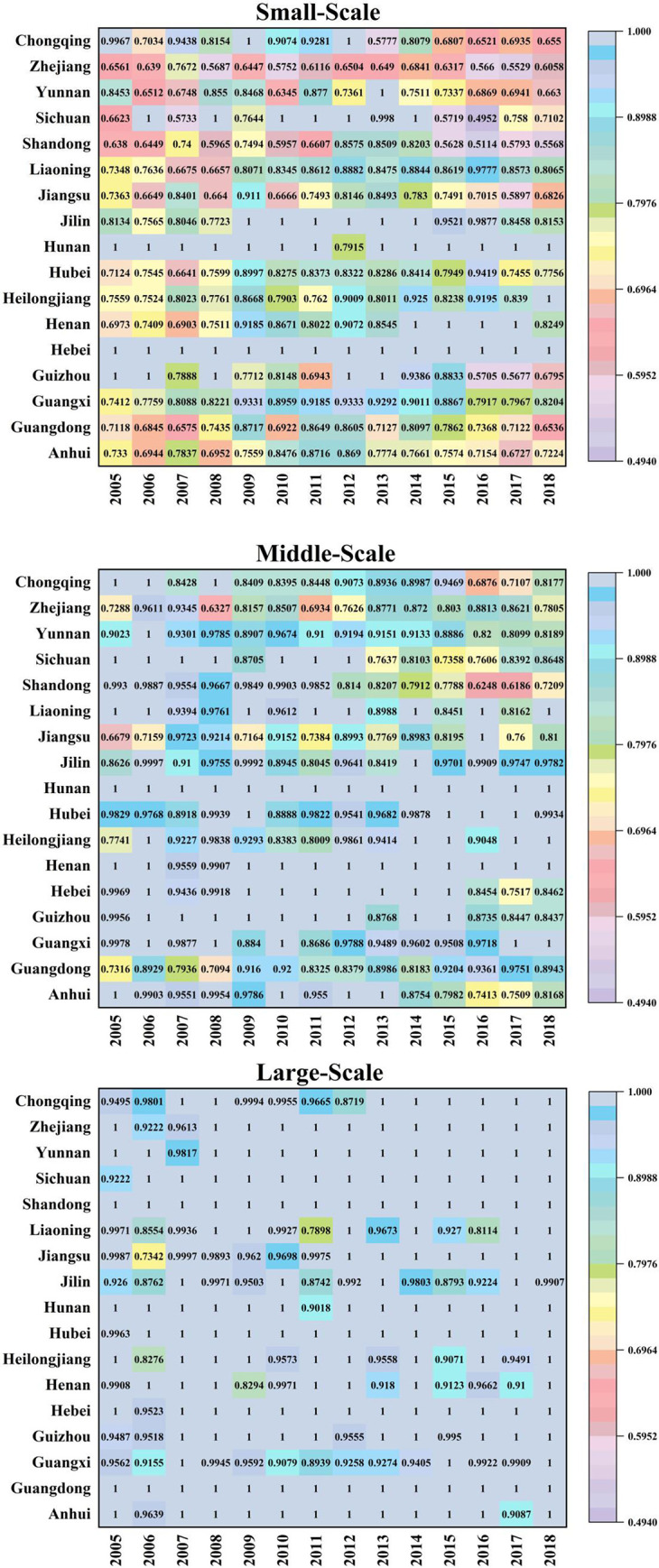
Three-sized TGR from 2004–2018.

As shown in Fig 5, the technology gap ratio is used to measure the distance between the optimal production scale and the potential optimal scale of the group. The closer the ratio close to 1, the closer the scale level gets to the optimal potential scale level, otherwise, it indicates the larger the gap between the scale level and the potential optimal scale level is. Based on the perspective of diverse breeding level, the TGR of common frontier and group frontier of different scale pig breeding is obtained by using common frontier theory. From the perspective of time trend, the TGRs of middle-scale and large-scale in most provinces were almost 1 every year, and by 2018, large-scale TGR was not 1 only in Jilin. Before 2012, the development of medium-sized pig breeding was good. However, TGR presented a trend of continuous diffusion in recent years, indicating that the development of medium-sized scale was not in line with the development trend of China’s pig breeding industry, thus either to expand the scale and step into large-scale pig breeding, or reduce the scale and change into farmers individual breeding mode. The small-scale TGR in Hebei is 1 every year, indicating that Hebei is more suitable for the development of small-scale pig breeding. In Hunan, small-scale TGR is not 1 only in 2012, medium-scale TGR is 1 every year, and large-scale TGR is not 1 only in 2011, indicating that the scale of hog breeding in Hunan is reasonable.

### 4.2 China’s spatial change of GTPB

The comprehensive GTPB of the three scales under the common frontier and the scale frontier is shown in Table 2. In general, GTPB under group front is higher than GTPB under common frontier. Spatially, GTPB is the highest in the western region, followed by the eastern region, and the lowest in the central region [61]. Under the meta-frontier, the GTPB of the western region, the central region and the eastern region is 0.9993, 0.9974 and 0.9981 respectively, and the group frontier are 1.0092, 1.0023 and 1.0047, respectively. The leading cause for the high level of GTPB in the western region is that the improvement of farm facilities and the application and popularization of information technology in the western region have greatly raised the GTPB. The central region is densely populated and is not suitable for the development of pig breeding industry due to regional conditions. The breeding technology and management are relatively backward in the central area, resulting in low efficiency of pig breeding environment efficiency. GTPB in the common frontier presents a downward trend, while under the group frontier presents an upward trend. This reveals the overall development of China’s pig breeding industry is relatively optimistic.

**Table 2:** Average GTPB in each province and three regions.

	Meta-frontier	Group-frontier
Guangdong	1.0246	1.0316
Hebei	0.9780	0.9906
Jiangsu	1.0131	1.0143
Liaoning	0.9866	0.9921
Shandong	0.9809	0.9845
Zhejiang	1.0051	1.0149
Eastern Average	0.9981	1.0047
Anhui	0.9907	1.0025
Henan	0.9921	0.9958
Heilongjiang	0.9866	0.9964
Hubei	1.0210	1.0279
Hunan	1.0204	1.0126
Jilin	0.9735	0.9785
Central Average	0.9974	1.0023
Guangxi	1.0020	1.0072
Guizhou	0.9633	0.9637
Sichuan	1.0184	1.0261
Yunnan	1.0054	1.0221
Chongqing	1.0075	1.0269
Western Average	0.9993	1.0092

From the perspective of a single province, under the common frontier, the GTPB of Guangdong (1.0246), Hubei (1.0210), Hunan (1.0204) and Sichuan (1.0184) is relatively high, while under the group frontier, the GTPB of Guangdong (1.0316), Hubei (1.0279), Chongqing (1.0269) and Sichuan (1.0261) is relatively high. Among the 17 provinces, Hunan ranks first in small-scale GTPB, while the medium-sized development level is general. Chongqing ranks first in large-scale GTPB, but the level of development in small-scale is average. It reflects that the development situation of each provinces will be slightly different considering the heterogeneity among diverse scales. Once again, this confirms that the feeding mode, management mode, pollution emission and pollutant treatment mode of different scale pig breeding are different. The existence of scale heterogeneity should be taken into account in the analysis of GTPB.

**Fig 6:**
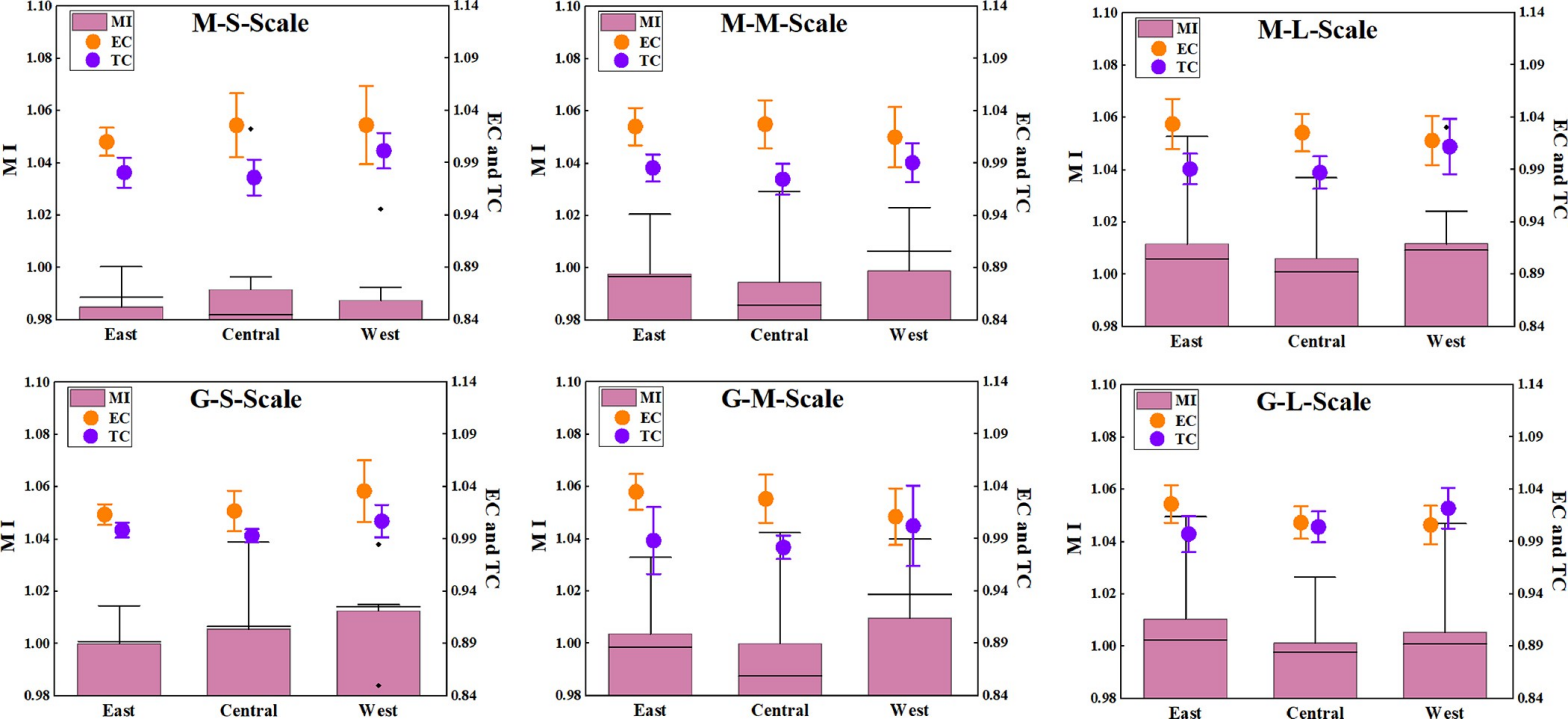
Three-sized average GTPB and its decomposition index in different regions.

The situation of various scales pig farming in three regions is shown in Fig 6. Under the common frontier, the regions with the highest GTPB sizes are the central region (0.9915), the western region (0.9988) and the western region (1.0117). Under the group frontier, the regions with the highest GTPB scale are the western region (1.0125), the western region (1.0097) and the eastern region (1.0104). Overall, the GTPB of the western region and eastern region is higher than that of the central region. Leading pig enterprises such as “Muyuan Group” and “Wenshi Group” are established in this region, resulting in excellent talents and advanced technology incline to the region. Besides, the western region has superior environmental conditions, with the dual characteristics of high fecal consumption capacity and low feed transportation cost. The central region is limited by geographical, resources breeding foundation and other conditions, which makes the comprehensive GTPB is low. Heilongjiang and Jilin belong to the type of low pollutant reduction and large-scale breeding. In these provinces, the pollutants have not been better treated, which hinders the elevation of efficiency significantly.

In general, there was a positive correlation between GTPB and breeding scale, that is, large-scale GTPB was higher than medium-sized, and medium-sized GTPB was higher than small-scale, except for some specific years. This feature is determined by the scale effect. The large-scale farms have relatively perfect pollution treatment equipment, feeding technology and management means. In addition, the higher degree of utilization of fecal resources can improve GTPB. However, the small-scale and medium-sized breeding pollution treatment equipment is not superb, the cost of fecal treatment is high, and the income is low, resulting in low GTPB.

Under the meta-frontier, the average GTPB of small-scale, medium-sized and large-scale in China is 0.9880, 0.9969 and 1.0097, the EC is 1.0196, 1.0221 and 1.0253 respectively, and the TC is 0.9851, 0.9832 and 0.9957 respectively. Under the group frontier, the average GTPB of the three scales is 1.0057, 1.0041 and 1.0057, the EC was 1.0204, 1.0246 and 1.0130, and the TC is 0.9991, 0.9900 and 1.0069 respectively. The results indicate efficiency improvement and technology retrogression. It is easy to find that China’s GTPB belongs to the mode of “technical efficiency induction + technological progress driven”, and there are significant characteristics in the development process, that is, there is almost no growth of technical efficiency and technological progress of pig breeding in three scales at the same time. That is to say, as one side promotes the growth of GTFP, it will always encounter the decline of the other side, which will have an adverse impact on GTPB. This phenomenon caused by the gap of factor constraints manifests that the promotion and diffusion of China’s pig breeding to the existing advanced green breeding technology is still unsuccessful, and the pig strategy cannot be separated from the traditional technology to the new environmental protection technology innovation.

**Fig 7:**
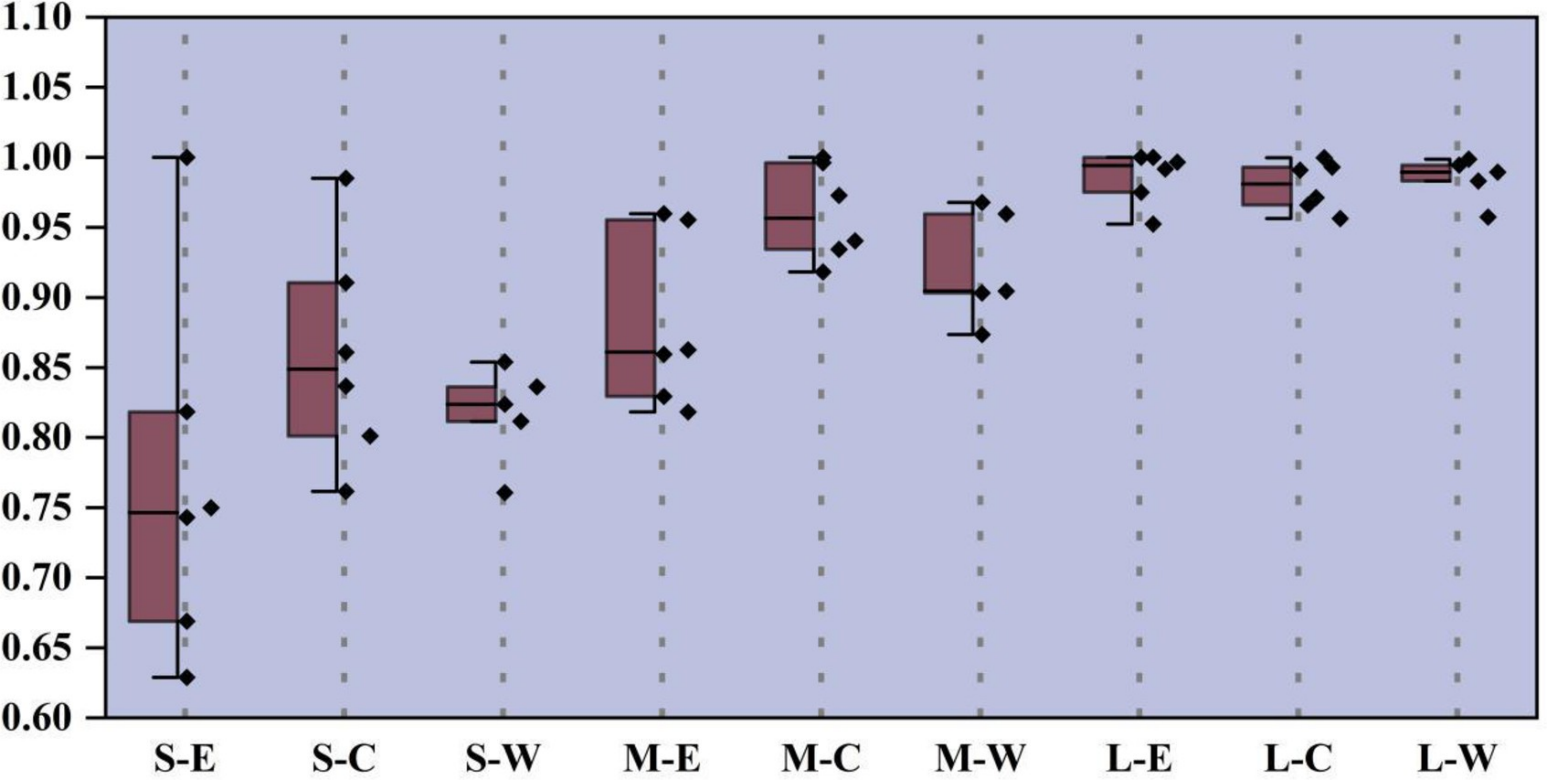
Three-scaled TGR in distinctive regions.

As shown in Fig 7, the average TGR of small-scale Eastern Areas, Central Areas and Western Areas is 0.7681, 0.8594, and 0.8173, respectively; the average TGR of medium-sized in three regions is 0.8808, 0.9604, and 0.9217, respectively; and the average TGR of large-scale is 0.9860, 0.9795, and 0.9846, respectively. The comprehensive TGR of large-scale pig breeding was highest (0.9834), followed by the middle scale (0.9210), and small scale (0.8149). It reveals the large-scale breeding has a high level of low-carbon technology. From the regional point of view, the TGRs in the eastern region (0.8783) and in the western region (0.9079) are relatively low, which demonstrates there is a big gap between the regions’ breeding technology and the optimal low-carbon environmental protection breeding technology, which does not reach the optimal state. This may be due to a series of reasons, such as extensive use of water, low level of technology, unreasonable farming structure and so on. As the main pig producing areas in China, the large-scale breeding in the five western provinces is in the rapid development stage, and the water environment pollution load is large, but the level of manure treatment technology and heating technology is lagging behind. Therefore, there should be a certain degree of resource preference for the region, and the key point is to strengthen the input of production factors and technical level of pig breeding in this region. Although the eastern region has a high level of technology, it has a dense population and limited pig breeding area, which does not achieve the optimal effect. Overall, the scale of farming has a huge impact on GTPB. The larger the scale, the greater the TGR.

## 5. Conclusions and policy implications

Based on the SBM model, this paper constructs the MML index considering negative output, measures China’s GTPB from 2004 to 2018, and draws the following conclusions:

1. From 2004 to 2018, China’s large-scale GTPB was the highest, followed by medium-sized GTPB and small-scale GTPB [62]. There is a close relationship between GTPB and breeding scale. The larger the scale is, the fuller the scale effect can be brought into play, making the growth of GTPB more advantageous. From the perspective of growth trend, the impact of environmental constraints on China’s pig breeding is greater, and the growth rate of GTPB is slowing down.
2. In terms of regional distribution, the western region is the highest, the eastern region is the second, and the central region is the lowest. It reveals that the unique geographical conditions are also very significant for the development of farming industry. It is necessary to develop pig industry on the basis of making full use of the existing natural conditions [8].
3. From 2004 to 2018, China’s GTPB shows efficiency growth and technology recession. The value of EC in three scales is greater than 1. The growth of GTPB in any scales depends more on the improvement of efficiency than the progress of technology. The pig breeding industry is generally fragile, which is greatly affected by emergencies [63]. The outbreak of blue ear disease in pigs in 2006, the global financial crisis in 2008, and the outbreak of influenza A (H1N1) virus in 2009 caused a heavy blow to the pig industry. Until 2011, the situation began to improve. The improvement of technical level and epidemic prevention level can reduce the harm to pig industry to a certain extent.
4. The TGR of large-scale pig breeding is closest to 1, followed by medium-sized, and finally small-scale, indicating that large-scale pig breeding is closest to the potential optimal technical environmental efficiency level, while the production technology level of small-scale pig breeding is relatively low. The results obtained under common frontier and group frontier are diverse. The GTPB under the common frontier is lower than that under the group frontier. The GTPBs of the three scales under the group frontier are all greater than 1, and the large-scale GTPB under the common frontier is greater than 1, showing a positive growth. The GTPB under the group frontier is obviously overestimated. Based on the above empirical results, we propose the following three policy recommendations:
  1. Vigorously develop large-scale pig farming, while coordinating the regional differences. Based on the spatial differences of China’s pig breeding, the government should give full play to the scale effect of large-scale pig breeding, and the optimal model of breeding with regional characteristics and manure discharge policy are reasonably formulated. Actively promote the exchange and cooperation of clean technology among provinces to ensure the effective spread and diffusion of advanced technology, so as to general upgrading and improvement of GTPB in all regions.
  2. In the process of breeding, it is necessary to continuously improve the level of low-carbon technology. On the basis of natural conditions, constantly perfect the breeding technology, and make the pig breeding scientific and rational. According to the different environment of each province, choose different varieties of breeding and fully promote the pig’s effective growth, so as to improve pig yield. Each region also needs to combine its actual situation, improve the scale and standardization degree, strictly control the fecal discharge and reduce the pollution pressure on the water environment.
  3. Pay attention to the related work of epidemic prevention. The occurrence of pig diseases and swine fever will cause a heavy blow to China’s pig breeding industry. Therefore, the key step is to strengthen the awareness of epidemic prevention of breeding personnel and make them understand the relevant epidemic prevention knowledge. The government should constantly enhance the propaganda of epidemic prevention work, improve the relevant epidemic prevention policies, so that breeders can comprehend the importance and harm of epidemic prevention. It can effectively raise the survival rate of pigs by avoiding the slackness of breeding personnel on epidemic prevention.

This paper is an considerable attempt to study China’s green total factor productivity of pig breeding from the perspective of scale. It can be used as a reference for the future research. However, the future work still has certain expansion space, such as study sample and time interval. Similarly, we also hope that this paper can promote more study in the direction of pig breeding, so as to design some policies to improve the productive efficiency of pig and ultimately reduce pollution emissions.
